# Whole exome sequencing reveals novel *LEPR* frameshift mutation in severely obese children from Western India

**DOI:** 10.1002/mgg3.692

**Published:** 2019-05-08

**Authors:** Arpan Bhatt, Charul Purani, Poonam Bhargava, Komal Patel, Tanvi Agarbattiwala, Apurvasinh Puvar, Krati Shah, Chaitanya G. Joshi, Nidhi Dhamecha, Mukund Prabhakar, Madhvi Joshi

**Affiliations:** ^1^ Department of Biotechnology Hemchandracharya North Gujarat University Patan Gujarat India; ^2^ Civil Hospital Ahmedabad Ahmedabad Gujarat India; ^3^ Gujarat Biotechnology Research Centre Gandhinagar Gujarat India; ^4^ ONE—Centre for Rheumatology and Genetics Vadodara Gujarat India

**Keywords:** consanguineous families, leptin receptor, morbid obesity, novel mutation, pathogenic mutation, severe early onset of obesity, whole exome sequencing

## Abstract

**Background:**

Obesity, especially early onset of obesity is a serious health concern in both developed and developing countries. This is further associated with serious comorbidities like a fatty liver disease, cardiovascular diseases, type‐2 diabetes, obstructive sleep apnea, renal complications and respiratory problems. Many times early onset of obesity is linked with heritable monogenic, polygenic and syndromic forms. Globally, studies on roles of genes involved in early onset of obesity are limited.

**Methods:**

Here in this study, a consanguineous family of Western Indian origin having four siblings, one unaffected and three affected with severe early onset of obesity was enrolled. Affected siblings also displayed comorbidities like mild to moderate obstructive sleep apnea, raised Renal Resistance Index, oliguria, and severe anemia. Whole Exome Sequencing (WES) of Trio with one affected and unaffected sibling was done. Data analysis was performed to check pathogenic mutation segregation in unaffected parents with affected and unaffected sibling.

**Results:**

WES of trio identified novel frameshift mutation in the *LEPR* gene resulting in truncated leptin receptor (LEPR). The same mutation was confirmed in other affected siblings and two siblings of distant relatives by Sanger sequencing. The possible effects of truncating mutation in LEPR function by in silico analysis were also studied.

**Conclusion:**

Understanding genetic basis of obesity might provide a clue for better management and treatment in times to come. This work demonstrates identification of novel mutation in *LEPR* gene resulting into early onset of obesity. Discovery of novel, population‐specific genomics markers will help population screening programs in creating base for possible therapeutic applications and prevention of this disease for next generations.

## 1 INTRODUCTION

Global data of obesity prevalence are based on the World Health Organisation (WHO) body mass index (BMI) cutoff value of 30 kg/m^2^ (Hunma et al., 2016). Moreover, BMI > 25 kg/m^2^ also puts at risk of diabetes (Riddle, 2018). Early onset of obesity is a serious and challenging issue in both developed and developing countries (Choquet & Meyre, 2010). It is increasing at an alarming rate with number of obese children rising almost 10‐fold from 11 to 124 million in a span of 40 years (WHO, 2018). Obesity has a high rate of heritability (>0.70) and the projected global burden increased to 1.12 billion people worldwide by 2030 (Kelly, Yang, Chen, Reynolds, & He, 2008; Walley, Blakemore, & Froguel, 2006).

Early onset of obesity in children is associated with comorbidities such as orthopedic, ophthalmologic, renal complications, obstructive sleep apnea, and other respiratory diseases (Choquet & Meyre, 2010). Furthermore, obesity with hyperphagia and onset at <5 years indicate genetic cause and heritability in patient's family (Farooqi & O'Rahilly, 2005). Early onset of obesity is associated with some of the Mendelian disorders like Bardet–Biedl syndrome, Prader–Willi syndrome, Alstrom and Cohen syndromes, etc. (Goldstone & Beales, 2008). Majority of early onset of obesity is linked with monogenic conditions. Researchers have identified genes of the leptin (LEP)–Melanocortin pathway associated with the monogenic forms of obesity by their effect on food intake and energy homeostasis (Huvenne, Dubern, Clément, & Poitou, 2016). In LEP‐Melanocortin pathway, LEP neurotransmitter passes blood–brain barrier and acts through arcuate nucleus of the hypothalamus (ARC). In ARC, it acts upon (a) appetite‐stimulant Neuropeptide Y and agouti‐related peptide neurons and (b) Anorectic pro‐opiomelanocortin (*POMC*) and cocaine‐ and amphetamine‐regulated transcript neurons (Paracchini, Pedotti, & Taioli, 2005). *POMC* product further gets cleaved and binds to melanocortin 3 and 4 (*MC3R*,* MC4R*) receptor. Impairment in this pathway leads to hyperphagia and early onset of obesity (Page, Shi, & Freemark, 2017). Activity of LEP is mainly dependent on selective binding with LEP receptor (LEPR). There are five isoforms of LEPR, where LEPR1 is the longest form. Animal studies have identified that LEPR1 is mostly expressed in hypothalamus and helps in transmission of LEP signal to the cells (Paracchini et al., 2005; Schwartz, Seeley, Campfield, Burn, & Baskin, 1996).

Rare and pathogenic variations in the *MC4R* (MIM:155541), *POMC* (MIM:176830), *LEP* (MIM:164160), *LEPR* (MIM:601007) and few other genes of LEP‐melanocortin pathway have been found and very less frequency of mutations was accounted in different studies due to complexity in the pathogenesis of obesity (Gill et al., 2014; Paracchini et al., 2005). Discovery of rare or novel mutations having a role in the pathogenesis of early onset of obesity by whole exome sequencing (WES) is a new approach in comparison to candidate gene approach and genome‐wide association studies.

This study was designed to identify an underlying genetic cause of severe obesity with occurrence in the first year of life in three siblings. Due to the complexity of this disease, WES was chosen to be the method of choice. The WES of trio (mother, father, and affected offspring) along with normal sibling was performed to identify pathogenic variation and its segregation amongst parents. This study also demonstrates the use of WES in the identification of novel mutations in consanguineous families exhibiting heritable monogenic disorders.

We report frameshift mutation of *LEPR* in severely obese children of the consanguineous family belonging to India using WES. To the authors knowledge, this is the first study from India reporting *LEPR* mutation in severe early onset of obesity children and parents. We also confirmed the same mutation through Sanger sequencing in two obese siblings of distant‐related consanguineous family.

## 2 MATERIALS AND METHODS

### 2.1 Ethical compliance

**Figure 1:**
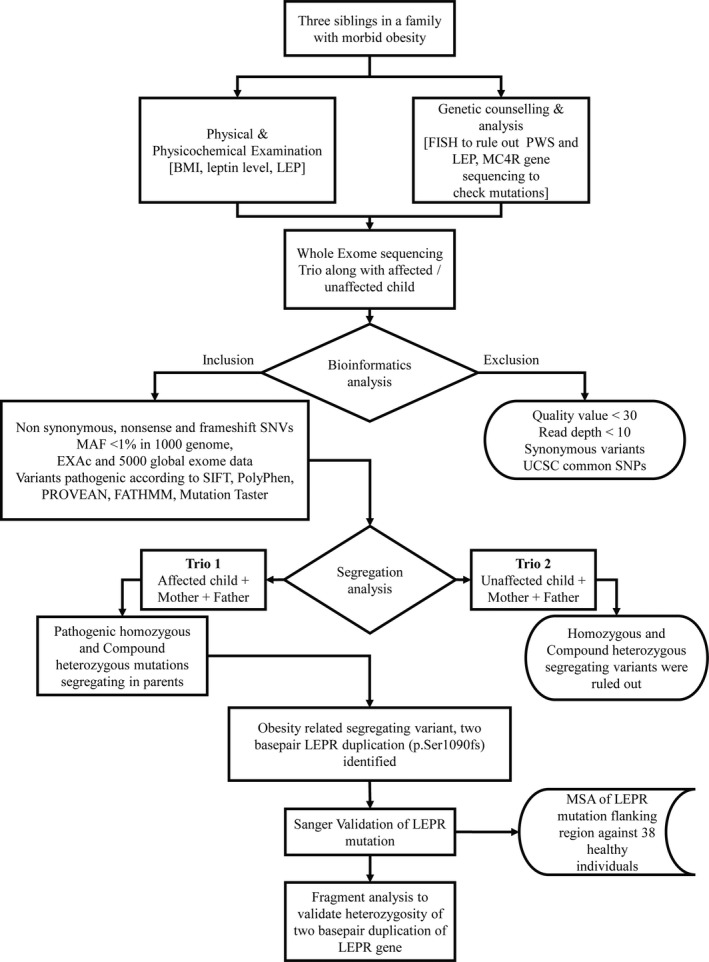
Schematic workflow depicting study design. BMI, body mass index; EXAC, Exome Aggregation Consortium; LEP, leptin; LEPR, leptin receptor; MAF, Minor Allelic frequency

This study (Figure 1) was approved by the institutional ethics committee of BJ Medical College, Ahmedabad and Helsinki's ethical principles were obeyed.

### 2.2 Subjects

Three severely obese children belonging to same consanguineous parents from Western India were recruited in this study. All three children were having BMI > 30 kg/m^2^ and occurrence after first year of life. Clinical evaluation was carried out at Pediatric department, Civil Hospital, Ahmedabad. In the later phase of study, two obese children (BMI > 30 kg/m^2^) belonging to consanguineous family (distantly related to the previous family) were recruited in this study. All subjects were hyperphagic with abnormally increased appetite.

Blood samples for DNA isolation were collected in K3‐EDTA from all family members including parents and grandparents. DNA was isolated using Purelink genomic DNA mini kit (Invitrogen, Thermo Fisher Scientific). Serum sample of subject MO1 was sent to Kasturba health society, medical research center, Mumbai, India to determine serum LEP concentration by ELISA method. The Fluorescence In Situ Hybridization (FISH) study was also done in Subject MO1 to rule out the possibility of Prader–Willi Syndrome.

### 2.3 Exome enrichment, library construction, and sequencing

In this study, we have sequenced exome of two siblings (one with early onset of obesity and other without any disease‐specific symptoms) and their parents. The initial amount of 100 ng of whole genomic DNA of each subject was amplified using Ampliseq RDY panel kit from Thermo Fisher Scientific. The panel encompasses ~294,000 primer pairs across 12 primer pools covering more than 97% of CCDS with 5 bp padding around exons. The library was prepared using Ion Ampliseq library kit plus (ThermoFisher scientific) and bar‐coded libraries were pooled in equimolar concentration to make the final concentration 500 ng. Library amplification and enrichment was done using Ion PI Hi‐Q OT2 200 kit (ThermoFisher scientific) on Ion OneTouch 2 and Ion OneTouch ES instruments, respectively. Libraries were then sequenced using Ion PI Hi‐Q sequencing 200 Kit on Ion Proton system according to the manufacturer's protocol.

### 2.4 Bioinformatics analysis

Sequencing reads passing quality value filter of 30 from Trio and unaffected sibling were aligned to the hg19 reference sequence using Torrent Suite v4.2.1 (Last accessed on October 1, 2018). Aligned BAM files were subjected to variant calling using Ion torrent inbuilt Torrent Variant Caller 3.6 Plug‐in. Variants were annotated using Ion Reporter V. 5.10.2.0 software (Last accessed on October 18, 2018). Variant analysis in Trio was performed to check the segregating mutation. We restricted our search for OMIM database genes due to disease relevance and quality check filters (quality value of ≥30 and read depth of at least 10) were applied. Homozygous or compound heterozygous SNVs and INDELs were in focus due to consanguinity. SNPs and INDELs with the Global Minor Allelic frequency of <1% according to the 1,000 genome project, Exome Aggregation Consortium and 5,000 Global exome data by NHLBI exome sequencing project were accounted. Nonsynonymous SNVs covering the CDS, UTR and splice site components of genes were checked for its pathogenic effect using pathogenicity prediction tools like SIFT (Ion Reporter), PolyPhen (Ion Reporter), PROVEAN (Choi & Chan, 2015), Mutation Taster (Schwarz, Cooper, Schuelke, & Seelow, 2014), FATHMM (Ion Reporter) and Grantham (Ion‐Reporter). Only, SNPs and INDELs which are pathogenic and segregating in the family were considered. Segregating mutations were compared with the unaffected sibling.

### 2.5 Fragment analysis

Heterozygosity of *LEPR* duplication in parents and grandparents was also confirmed by fragment analysis assay. A same forward primer which was used to validate mutation by Sanger's sequencing was end labeled with 6‐FAM dye and used for PCR amplification of gDNA. Products were run in 3500 XL capillary electrophoresis machine (Applied Biosystems) with LIZ‐250‐500 (Applied Biosystems) size standard. Fragments were analyzed in GeneMapper software version 4.2. Peak height of each sample was calculated to differentiate normal and mutated alleles.

### 2.6 Sanger validation

The coding region of the *LEP* (RefSeq: NG_007450.1) gene and *MC4R* (RefSeq: NG_016441.1) gene was screened in a subject MO1 prior to exome sequencing for SNPs. Postpurification PCR products were directly sequenced using Bigdye V.3.1 terminator chemistry. Sequences were analyzed in Codon code software and screened for variants in comparison with RefSeq NCBI database.

The mutation identified by exome sequencing in the Trio was validated by Sanger sequencing. Primers were designed flanking the mutation site (sequences will be available on request). Sanger Sequencing was done for two healthy controls and for all the family members to validate the mutation. Later, the same mutation was screened in two severe obese children from another consanguineous family who are distant relatives of Trio screened by exome sequencing.

DNA sequence flanking *LEPR* mutation was extracted from 38 nonobese individuals’ in‐house WES data. All the sequences were taken for multiple alignments in reference with subject's Sanger sequence data in Codoncode aligner. Purpose of multiple alignments of sequences extracted from WES data of healthy individuals with Sanger sequencing data was to calculate mutation frequency.

## 3 RESULTS

### 3.1 Clinical history

**Figure 2:**
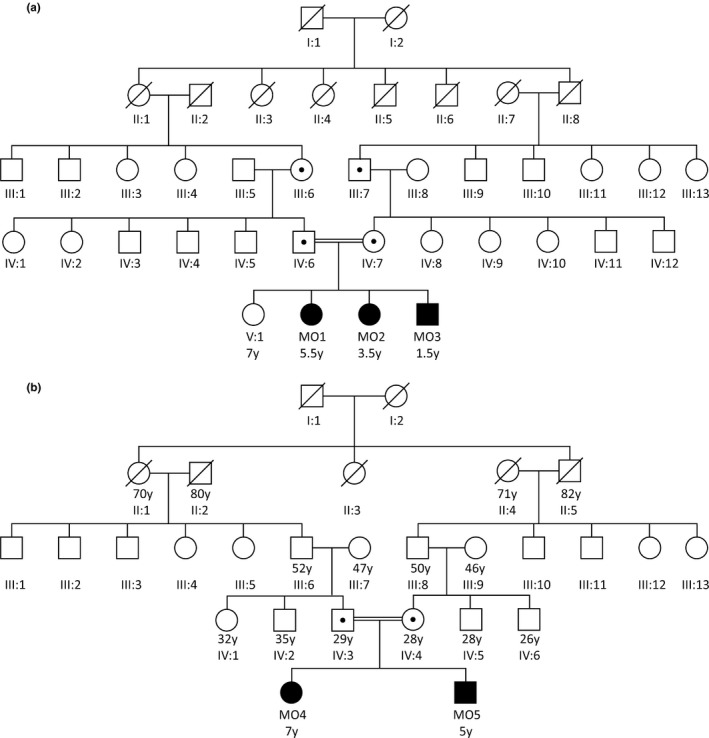
(a) Pedigree of Consanguineous family with three siblings (Subject MO1, MO2, MO3) affected. (b) Pedigree of Consanguineous family with two siblings (Subject MO4, MO5). Autosomal Recessive pattern of inheritance was confirmed by identifying heterozygosity of same *LEPR* mutation and shown by dot inside circle and square

Subjects MO1, MO2, and MO3 were born to third degree consanguineous parents from Western India (Figure 2a). Elder sibling (7 year, female) is unaffected, while the other three subjects are severely obese. Two siblings MO4 and MO5 are also born to third degree consanguineous parents from Western India (Figure 2b). Both families are related to each other with more than third degree consanguinity.

Subjects MO1, MO2, and MO3 were recruited with a complaint of extreme body weight, difficulty in walking, excessive and uncontrolled food intake, and hyperphagia. BMI index of all five patients was above the 97th percentile according to WHO child growth standards (birth–60 months) and WHO reference 2007 (61 months–19 years).

Subject MO1 is 5 years and 7 months old female, she started gaining weight after the sixth month, her birth weight was normal. Her sleep study showed mild obstructive sleep apnea. Raised Renal Resistance Index in the right kidney, oliguria, and hyperammonemia evidenced renal abnormality. Insulin level was in normal range while postprandial blood sugar was 174 mg/dl. Her Hb1A (6.1%) and fasting blood sugar (101 mg/dl) indicate pre‐diabetes. She showed severe anemia, Vitamin D3 insufficiency with, low serum iron and ferritin levels. Subject MO2 is 3 years and 7 months old female and started gaining weight at the same age as a subject MO1. Her sleep study showed moderate obstructive sleep apnea. Raised renal cortical echogenicity and oliguria was evidenced in this subject. Her bone age is similar to children of the age group 5 years and showed hypotonia on muscle charting. Ultrasonography of the abdomen revealed hepatosplenomegaly. Her fasting blood sugar was 139 mg/dl.

Subject MO3 is 1 year and 7 months old male. He started gaining weight like other two siblings after 5–6 months. He suffers from severe anemia but unlike the other two siblings, polysomnography test is normal. His 2D Echo test is normal but the BERA study showed unilateral severe to profound hearing loss.

Subjects MO4 and MO5 both suffer from somewhat similar problems like other three patients in terms of difficulty in walking due to overweight, breathing problem while sleeping, and hyperphagia. MO4 and MO5 also started gaining weight at 5–6 months of age.

### 3.2 Exome analysis

Ion PI chip has generated total 26.7 G bases in two runs from exome sequencing of Trio (Affected, Mother and Father) and normal sibling with mean read length of 166 bp. More than 93% of the bases were read at 20× coverage while 52.73% bases called at 100× coverage and average base coverage depth was 104.6 for total 2, 93,903 amplicons.

IonReporter V. 5.10.2.0 software's trio exome analysis workflow called total 77,691 variants in the Trio. All inclusion and exclusion analysis criteria filters were applied and resulted in 7,326 variants of 5,387 genes. All these variants checked for segregation among both the parents and as a result, five homozygous and three compound heterozygous mutations in the subject were identified as having pathogenic effect and segregating among parents.

**Figure 3:**
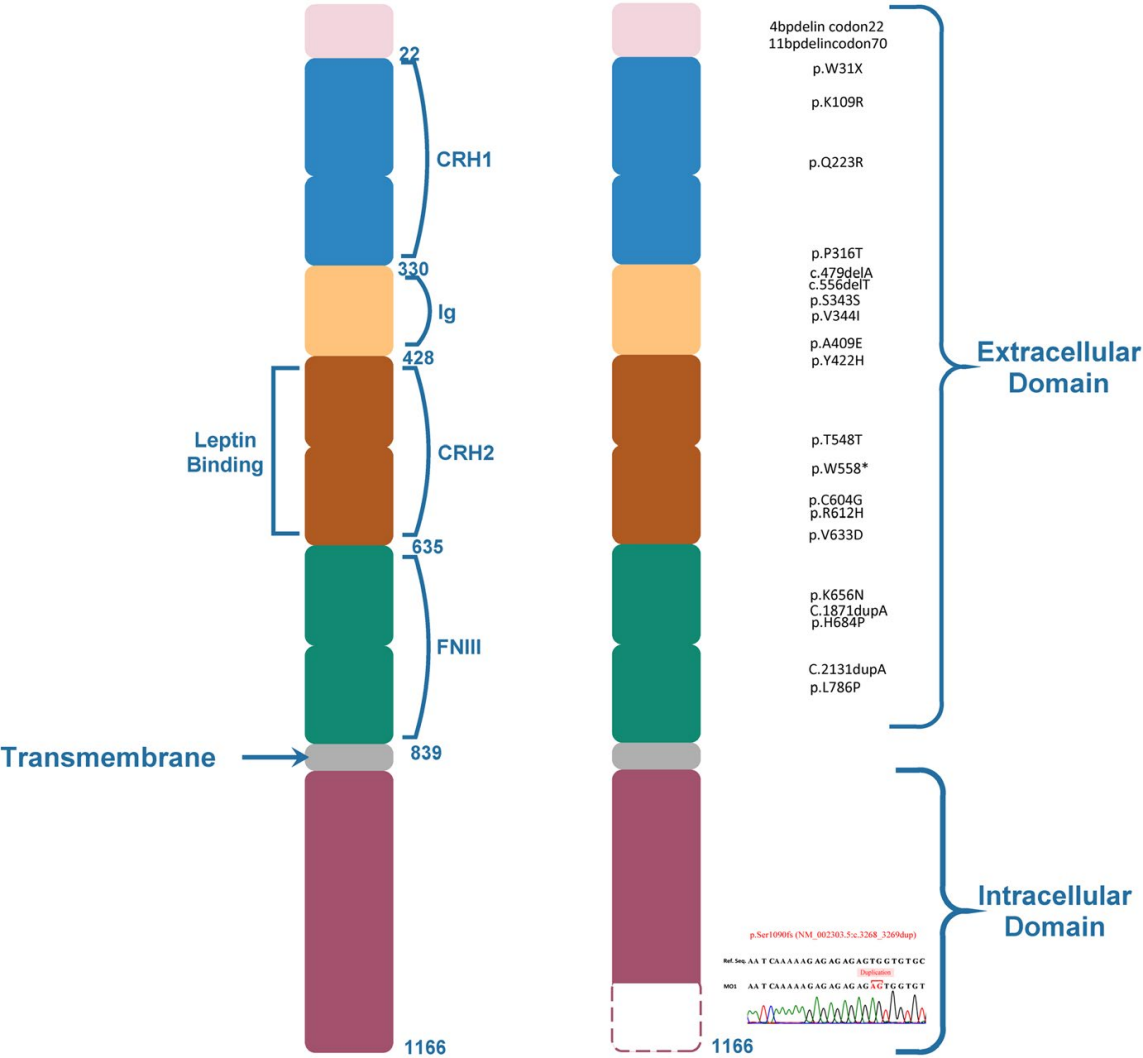
*LEPR* schematic protein structure depicting 71 amino acid truncation in the intracellular domain. Known mutations in *LEPR* were shown

A two base pair frameshift *LEPR* duplication, p.Ser1090fs (NM_002303.5:c.3268_3269dup) was the only mutation found related to subject's clinical manifestation. Same *LEPR* frameshift mutation was found in parents in heterozygous state. *LEPR* mutation found in the patients was in the intracellular domain of the protein. Frameshift mutation inserts stop codon in protein at 1,095 position as compared to 1,166 in wild‐type, leads to the translation of truncated protein (Figure 3).

### 3.3 Mutation validation

**Figure 4:**
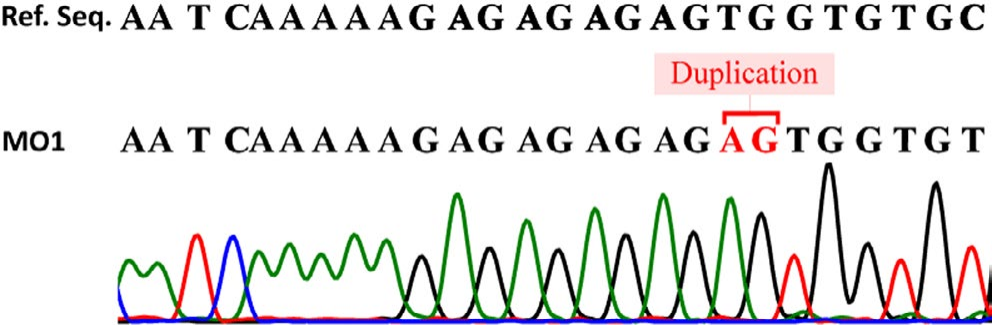
Sequencing result of *LEPR* region for the conformation of two base pair homozygous AG duplication

**Figure 5:**
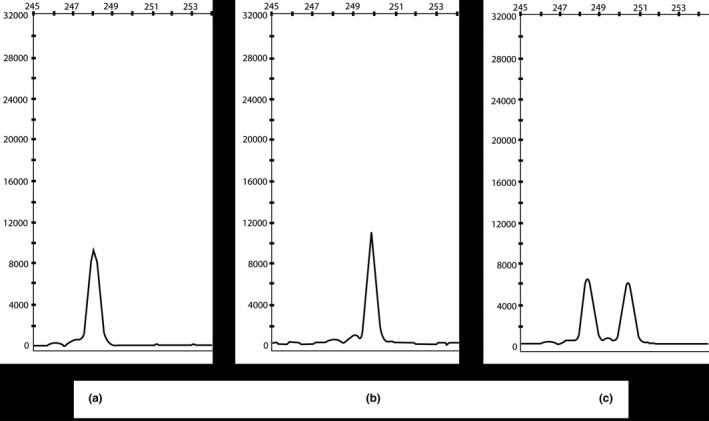
Fragment analysis result of *LEPR* fragment amplified covering *LEPR* p. Ser1090fs (c.3268_3269dup). Fragment analysis generated 248 bp homozygous fragment in Control individual (a), After duplication of 2 bp (AG), a homozygous fragment of 250 bp was identified (b), while in parental line presence of heterozygosity was confirmed by identification of both 248 bp (normal allele) and 250 bp (mutated allele) fragments

Homozygosity of p.Ser1090fs *LEPR* mutation was confirmed in three siblings (Subject MO1, MO2, and MO3) and cousins (Subject MO4 and MO5) using Sanger's dideoxy method (Figure 4). Sequencing showed the absence of same *LEPR* mutation in elder sibling of subject MO1, MO2, and MO3 with normal phenotype and other healthy controls. The mutation identified in five subjects was submitted to ClinVar database (accession number: SCV000622177). Heterozygosity of same *LEPR* mutation was confirmed using fragment analysis assay in Mother, Father, paternal grandmother, and maternal grandfather (Figure 5).

**Figure 6:**
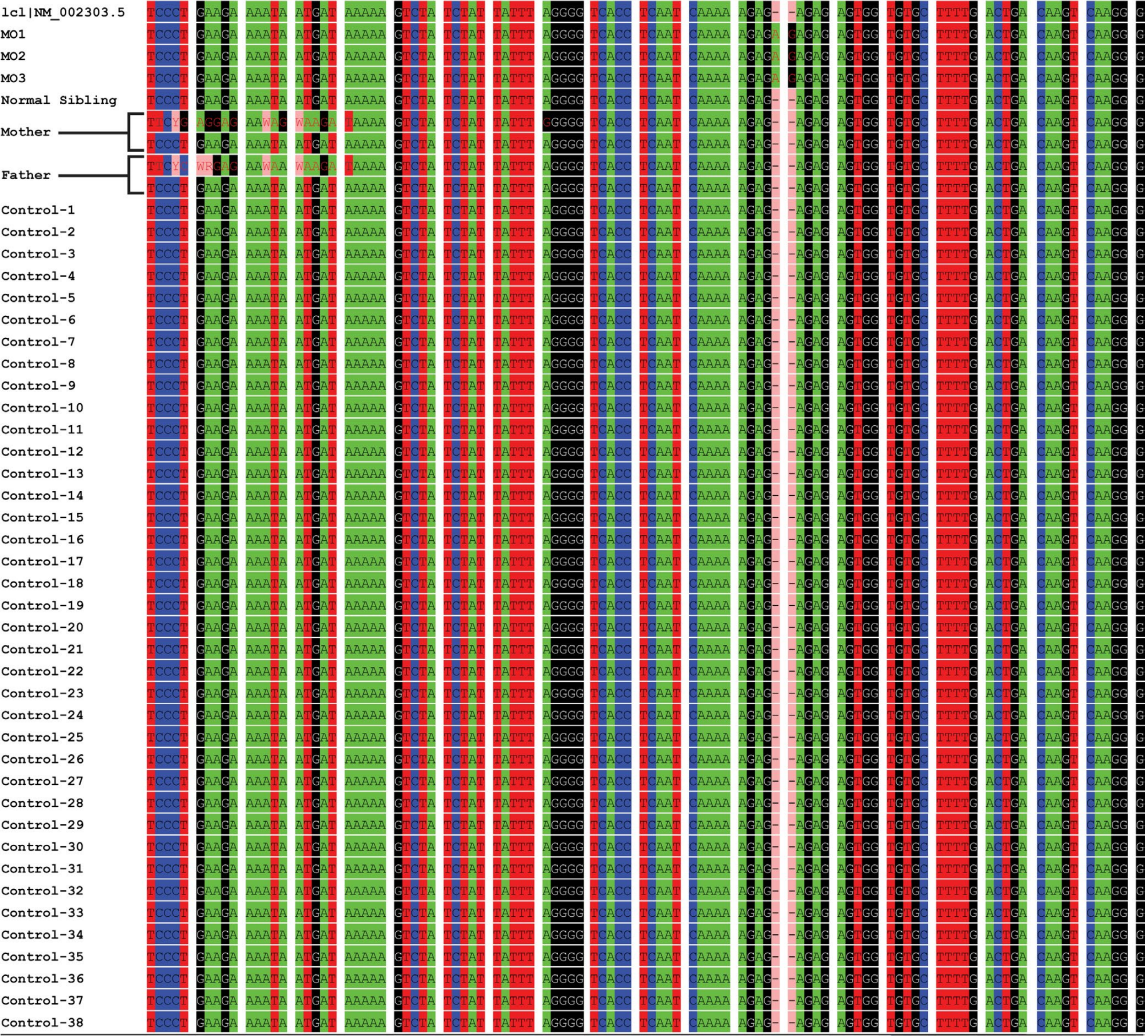
Multiple alignment of sequences covering p. Ser1090 fs (c.3268_3269dup) variation of 38 healthy controls along with affected subjects and parents

Sequence alignment data from 40 nonobese individuals in reference to affected subjects showed a complete absence of two base pair duplication in the *LEPR* gene. Hence, the calculated frequency of NM_002303.5:c.3268_3269dup in nonobese West Indian population was zero (Figure 6).

## 4 DISCUSSION

Obesity is caused generally due to disturbances of balance between food intake and energy expenditure, which is because of disruption of signaling which regulates complex body system including hunger and lipid as well sugar metabolism (Bell, Walley, & Froguel, 2005). Other than known syndromes associated with obesity, there are multiple monogenic forms of obesity which include *LEP*,* LEPR*, *MC4R*, and *POMC1* majorly.

LEP is a cytokine‐based hormone, which is primarily secreted by adipose tissue, helps to maintain energy homeostasis and neuroendocrine function through LEPR (Bates & Myers, 2003). Lack of LEP‐mediated signaling (mutation in *LEP* or *LEPR*) leads to the increased tendency of food uptake, severe obesity, hypothyroidism, and infertility (Bates & Myers, 2003; Huang & Li, 2000).

**Figure 7:**
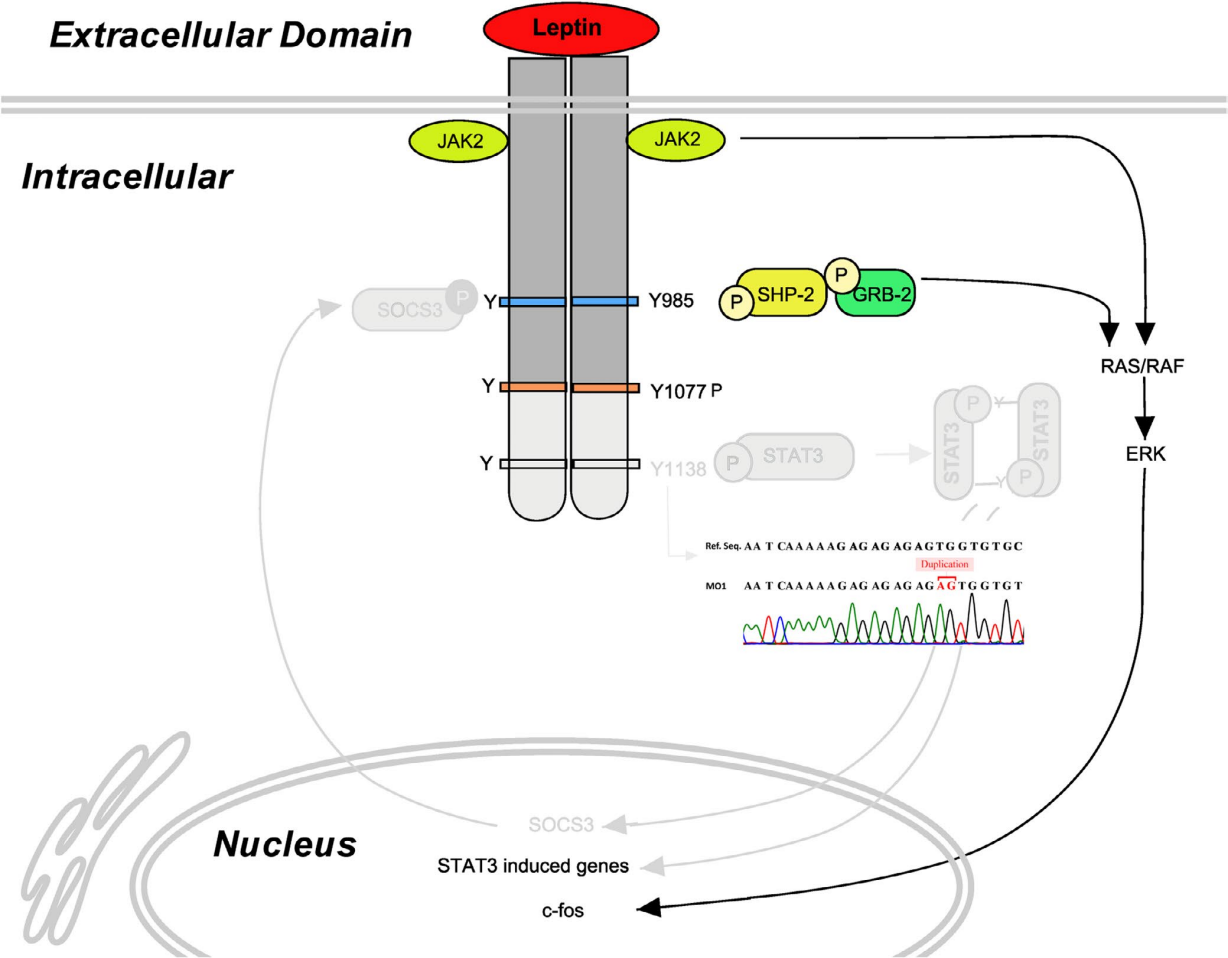
Showing Possible effect of the mutation in leptin–leptin receptor signaling pathway

Longest and highly conserved isoform of LEPR is B (LRb) which is from class I family of cytokine receptors (Buettner et al., 2006; Tartaglia et al., 1995). LEP binds to LRb and activates Janus kinase (JAK2) and triggers phosphorylation of cytoplasmic target proteins (Buettner et al., 2006; Vaisse et al., 1996). Binding of LEP to LRb induces homodimer formation of LRb, which further activates associated JAK2, which further phosphorylates Tyr985 and Tyr1138 of the intracellular domain of the receptor. Phosphorylated Tyr985 activates ERK signaling pathway in downstream and phosphorylated Tyr1138 recruits STAT3 protein to receptor complex and phosphorylates it. Tyrosine phosphorylated STAT3 subsequently translocates to the nucleus and mediates transcriptional activation of many genes. This also includes the gene for a member of the suppressors of the cytokine signaling family (SOCS3) which negatively inhibits LEP feedback pathway (Figure 7) (Bates & Myers, 2003, 2004; Yang & Barouch, 2007).

In this study, we found novel frameshift *LEPR* mutation in a consanguineous family with three children with hyperphagic early onset of obesity. This frameshift two base pair duplication generated truncated LEPR (pSer1090fsX5). The previous study showed mice carrying a *LEPR* gene mutation for Tyr1138 (s/s mice) are unable for the activation of STAT3 by LEP and lack the ability of LEP to signal through STAT3. Inactivation of STAT3 signaling nullifies LEP activity, which leads to the hyperphagic condition (Buettner et al., 2006). Circulating serum LEP level of subject MO1 was above the range of normal, showing LEP resistance (Table 1). Hypogonadism was not possible to check because of the small age of all three patients, however, the advanced bone age of 5–6 years of subject MO2 at 3 years and 7 months was indicative of the development of some secondary characters. Other members of the family carrying heterozygous *LEPR* mutation were not showing any phenotypic resemblance to the patients.

**Table 1:** Anthropomatric measurement of patients with their age

Sr. No.	Proband	Gender	Age	Height (cm)	Weight (kg)	BMI (kg/m^2^)	Leptin (ng/ml)
1	MO1	F	5 years 7 months	91	33.5	41.5	24
2	MO2	F	3 years 7 months	107	47.5	41.5	**—**
3	MO3	M	1 year 7 months	71	16	31.1	**—**
4	MO4	F	6 year 7 months	139	58	30	**—**
5	MO5	M	5 years	117	51	37.7	**—**

Notes

Leptin measurement of Patient MO1 is indicated.

BMI, body mass index.

In previous studies, occurrence of *LEPR* mutation in a cohort of 39 children of consanguineous origin was reported to be 3% (Saeed et al., 2014), compared to the occurrence of *LEPR* mutation in four consanguineous families with severe obesity at 50% (Gill et al., 2014). In this study, we focused on two consanguineous families related to each other helped us in digging for novel autosomal recessive LEPR mutation.

Advancement in LEP or LEPR therapeutics gives many options to overcome LEP or LEP resistance. LEP sensitizing molecules, LEPR agonists, alleviating the inhibition of the LEPR signaling, increasing the pool of surface receptors accessible to LEP, increasing LEP transport through the blood–brain barrier and decreasing ER stress with chemical chaperones are options available nowadays (Roujeau, Jockers, & Dam, 2014).

## 5 CONCLUSION

In summary, our study has identified a novel frameshift *LEPR* mutation in severely early onset of obesity children belonging to consanguineous family from Indian subcontinent. It suggests extensive use of WES technique over single gene screening techniques for variant discovery in families with such extreme phenotypes. In future, region‐ or society‐specific extreme sampling for such phenotype may lead us to the discovery of founder mutation.

## AUTHOR CONTRIBUTIONS

AB and MJ have participated in conceptualization, designing, experimentation, data analysis, and manuscript preparation. PB has participated in experimentation and manuscript drafting. KP and TA have participated in experimentation and data analysis. CJ has contributed in data analysis. AP has contributed in data analysis and data acquisition. CP, KS, ND and MP have participated in clinical workup and data acquisition. All the authors have read and agreed to the final manuscript.

## CONFLICT OF INTEREST

There is no conflict of interest.
